# The Numerical Analysis of the In-Plane Constraint Influence on the Behavior of the Crack Subjected to Cyclic Loading

**DOI:** 10.3390/ma14071764

**Published:** 2021-04-02

**Authors:** Jaroslaw Galkiewicz, Urszula Janus-Galkiewicz

**Affiliations:** Faculty of Mechatronics and Mechanical Engineering, Kielce University of Technology, al. Tysiąclecia Państwa Polskiego 7, 25-314 Kielce, Poland; ujanus@tu.kielce.pl

**Keywords:** fatigue, fatigue crack growth, in-plane constraints, T-stress, modified boundary layer model approach

## Abstract

The paper presents the influence of in-plane constraints defined by T-stress on the behavior of a crack subjected to cyclic loading. In the analysis, a modified boundary layer model approach was used in which the cohesive model was introduced. In the simulations, the constant maximum value of the stress intensity factor and four levels of T-stress were defined. The model was subjected to ten repeated stress cycles. Based on the results obtained, an analysis of the effect of the in-plane constraint on selected aspects of crack behavior was made. The strong influence of in-plane constraint applied in the model on the crack closure and the fatigue crack growth rate was proven. Since the in-plane constraint described the influence of geometry on the stress field surrounding the fatigue crack tip in real geometry, the results suggested that it is possible to create precise formulae connecting the level of the in-plane constraint with the effective stress intensity factor range and to incorporate the T-stress or Q-stress level in the Paris law.

## 1. Introduction

The influence of element geometry on crack behavior has been a problem from the very beginning of research on fracture toughness and fatigue. Its practical expressions are the sharp restrictions placed on the specimen geometry for testing, with relevant standards [1,2]. Therefore, the scientific community dealing with the topic is searching for a parameter that would mathematically capture the impact of the dimensions and in-plane geometry of the element used in research on the results.

Larsson and Carlsson were the first to propose a parameter allowing to estimate the influence of in-plane constraints, i.e., the shape and size of the specimen [3]. They proved that the second term of Williams’ asymptotic expansion [4,5]—the T-stress, which depends on the in-plane dimensions of the specimen and affects the shape and size of the plastic zone, and thus the value of the J-integral. Similar in tone were the works presented by Hancock et al. [6,7,8]. They paved the way for searching the relationship between geometry and fracture toughness. It is reasonable to assume that the T-stress associated with the shape and in-plane dimensions of the element, i.e., the so-called in-plane constraint, will affect the behavior of the fatigue crack.

In 1970, W. Elber [9] noticed that due to the action of a particular tensile stress field in the zone behind the increasing fatigue crack, the crack closed, and it was open only for a part of the loading cycle which affected the rate of crack growth. In the literature on fatigue, many articles analyzing the parameters affecting crack growth and closure can be found. The effects of load amplitude and stress ratio are the easiest to notice, e.g., [10,11,12]. The best example of such an analysis is the Paris law [13], in which the crack growth strictly depends on the value of the stress intensity factor (SIF) and the stress ratio R. It is much more difficult to describe the influence of the geometrical quantities characterizing the specimens used. In [10] the effect of specimen thickness was taken into account, in [11] the effect of crack length was taken into account, and in [14] the crack closure was analyzed in the case of a quarter-elliptical corner crack. These articles showed that, while taking into account the effect of the load was not a problem, the impact of geometry has not come to a coherent concept. The use of specimen characteristic dimensions is troublesome because it can only be used with a limited geometry class.

From Da Vinci’s time, it has been clear that the strength of a part subjected to static load depends on its size [15]. In fatigue analyses, this fact is even more pronounced. That is proved by the procedures presented in [16,17]. The methods for fatigue strength assessment include both the size of elements and the notch effect (shape of the element). In more recent works [18,19], the shape and size of specimens were also introduced to fatigue life assessment by a statistical approach. The concepts of highly stressed volume (HSV) and Weibull distribution were utilized. Results clearly indicated that the geometry of the specimen influenced S–N curves. In the review paper [20], two statistical approaches were also presented; the critical defect method and the weakest link method (Weibull model). In the paper [21], to describe the fatigue crack growth, a Dugdale strip yield model was included together with the crack closure effect; however, the authors were concentrated on the scattering of results for a single geometry and did not analyze the parameters that may influence the crack closure.

**Figure 1:**
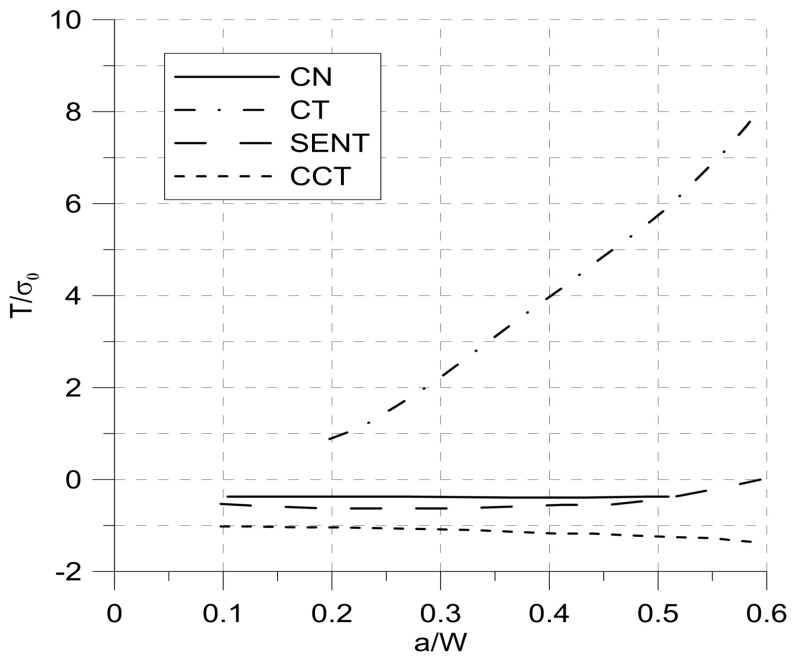
Dependence of T-stress on the geometry of the samples, where a—crack length, W—sample width, CN—corner notched specimen, CT—compact tension specimen, SENT—side edge notched tension specimen, CCT—center cracked tension specimen.

In contrast to previously cited papers in [22] the authors tried to explain the influence of geometry on fatigue behavior in a low-cycle range with stress triaxiality and Lode parameter. Application of parameters describing the state of stress instead of a probabilistic approach was a promising direction since in fatigue analyses there were no attempts to introduce a parameter that could quantitatively describe the influence of the size and shape of the applied specimen. It seemed that T-stress describing stress triaxiality could be a good candidate. Its value depended on the dimensions of the loaded element and the length of the crack (Figure 1) [23]. The T-stress in Figure 1 is normalized by the yield stress σ_0_.

One of the necessary tools to analyze crack growth is numerical analysis. However, apart from the first method, the simple node release technique [24] or virtual crack extension method utilized until now, many new sophisticated methods were developed that were more suitable for the specific applications. Contemporary development helped to solve almost every problem. In [25], a moving mesh technique was used for interlaminar damage in layered structures. The main advantage of the new approach was the decrease of the cost of computation due to the efficient decreasing of the time of computation compared to the classical cohesive zone model approach. In the case of mixed mode, the main problem was a prediction of the crack path. This prevented from designing a suitable mesh surrounding the crack tip. Finding the new direction of a crack increase is a strenuous, time-consuming procedure requiring the rebuilding of the mesh in the neighborhood of a crack tip. The two-stage method to model crack growth was presented in [26]. The clue of the method was the splitting of quadrilateral elements in the crack tip into triangular elements. This procedure can help to avoid problems with mesh tangling that can happen in other approaches. New methods to model the crack increase presented in [27] helped to shorten the time of computation by proposing a new procedure for predicting crack growth that reduced the number of re-meshing stages. Paper [27] utilized an arbitrary Lagrangian–Eulerian formulation to the static load problem; however, it was also an effective tool in the modeling of dynamic crack growth [28]. An interesting phenomenon that could happen during crack growth is branching. New problems arose in this case since the conditions for branching had to be established, the directions of growth of two new cracks had to be predicted, and the speed of the cracks had to be determined, to mention the most important. In [29], the authors improved a well-known method, XFEM, introduced in the beginning of the 21st century to solve problems of arbitrary crack propagation by introducing enriched shape functions. Thanks to that, they were able to model elements that were crossed by two cracks. In the paper, a modified boundary layer approach (MBLA) was used to describe the impact of the T-stress on the crack behavior during cyclic loading. In this case, the load was applied slowly, and the crack path was predictable. That allows for modeling crack growth by a cohesive zone model. MBLA made it easy to design a load in which only the T-stress value changed for the selected value of SIF.

The paper is organized as follows. Section 2 describes the solution for the crack tip stress field in elastic material, then a modified boundary layer approach is explained. In Section 4 and Section 5, the details of the numerical model are explained, Section 6 provides the test of numerical results, and then the results of the computation and conclusions are presented.

## 2. Williams Asymptotic Solution

In 1952, M. L. Williams proposed a solution for the stress field around the crack tip in the elastic material using the Airy function. He obtained a general formula for stresses and displacements in the Equation (1):

(1) $$U=\sum_{n=1,3,5\ldots }r^{1+\frac{n}{2}}\left\{C_{1n}\left(\cos\frac{n-2}{2}\theta -\frac{n-2}{n+2}\cos\frac{n+2}{2}\theta \right)\right\}+\sum_{n=2,4,6\ldots }r^{1+\frac{n}{2}}\left\{C_{1n}\left(\cos\frac{n-2}{2}\theta -\cos\frac{n+2}{2}\theta \right)\right\}\;\sigma_{rr}=\sum_{n=1,2}r^{\lambda -1}C_{1}\left[\left(-\lambda^{2}+3\lambda \right)\cos\left(\lambda -1\right)\theta +\left(\lambda^{2}-\lambda \right)\frac{\sin\left(\lambda -1\right)\pi }{\sin\left(\lambda +1\right)\pi }\cos\left(\lambda +1\right)\theta \right]\;\sigma_{\theta \theta }=\sum_{n=1,2}\left(\lambda^{2}+\lambda \right)r^{\lambda -1}C_{1}\left[\cos\left(\lambda -1\right)\theta +\frac{\left(\lambda -1\right)\sin\left(\lambda -1\right)\pi }{\left(\lambda +1\right)\sin\left(\lambda +1\right)\pi }\cos\left(\lambda +1\right)\theta \right]\;\sigma_{r\theta }=\sum_{i=1,2}r^{\lambda -1}C_{1}\left[\left(\lambda^{2}-\lambda \right)\sin\left(\lambda -1\right)\theta +\frac{\left(\lambda +\lambda^{2}\right)\sin\left(\lambda -1\right)\pi }{\sin\left(\lambda +1\right)\pi }\sin\left(\lambda +1\right)\theta \right]$$

where *λ* (*λ* = *n*/2) is the exponent of the Airy function; *n* = 1, 2, *θ*, and *r* are the polar coordinates of the coordinate system located at the crack tip; and *Cs* are numerical factors.

The function describing the stress distribution after transformation and simplification takes the Equation (2):

(2) $$\sigma_{ij}=Kr^{-\frac{1}{2}}f_{ij}^{1}\left(\theta \right)+A_{2}r^{0}f_{ij}^{2}\left(\theta \right)+A_{3}r^{\frac{1}{2}}f_{ij}^{3}\left(\theta \right)+\ldots$$

where: *K* is the stress intensity factor, *As* are higher-order coefficients (*A*_2_ is T-stress), and *fs* are functions describing the tangential changes of the stress tensor components.

Assuming that the plastic field surrounding the crack tip is small, the first element of the asymptotic expansion (2) is dominant, and, therefore, the higher-order members could be neglected. However, it has been noticed since the work of Larsson and Carlsson [3] that analyses based on a singular term of the asymptotic solution, are erroneous and a two-parameter description should be used when assessing fracture toughness. This theory was also transferred to elastic-plastic materials, in which the approach proposed by O’Dowd and Shih [30,31] using the Q parameter turned out to be the most popular.

## 3. Modified Boundary Layer Approach

For testing the crack tip behavior in elastic-plastic material under load without taking into account the finite geometry, the boundary layer approach is a very good solution. The boundary layer approach model is a disc-shaped, plane strain finite element model. In the model, a crack was introduced, and the crack tip was located in the center of the disc. The boundary layer approach model involved the loading of the external circular edge with displacements described by Equation (1). The basic condition for obtaining the correct results was to choose the load so that the plastic zone was small compared to the outer radius of the semi-circle. In the case of monotonic loading, one could take advantage of the symmetry and model only a semi-disc. The model in which the second term of the asymptotic expansion is included in the load is called the modified boundary layer approach [32,33], and it allowed for consideration of the influence of geometry.

## 4. Numerical Model

**Figure 2:**
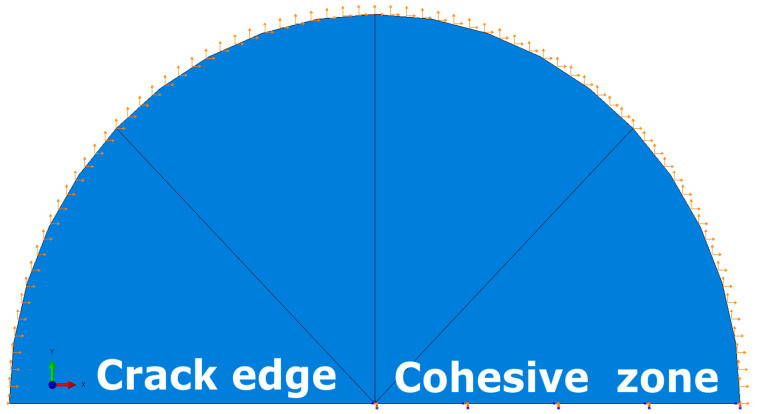
Boundary conditions.

The model’s geometry was a semi-circle (Figure 2). The outer radius of the semi-circle indirectly depended on the value of the load, and directly on the size of the plastic zone, which must be small compared to the radius [34]. In the analyzed problem, the external radius was 2 meters. The crack tip was located in the center of the circle. On the external edge of the model, displacements resulting from the applied SIF value were applied, which in each case was equal to 106 MPa√m. For the material properties defined in Section 5, this value was equivalent to the J-integral equal 50 N/m according to Equation (3), valid for the plane strain (PS):

(3) $$J_{I}=K_{I}^{2}\left(\frac{1-\nu^{2}}{E}\right)$$

where: *K* is the stress intensity factor, *ν* is Poisson’s ratio, *E* is Young’s modulus.

In the case of the modified boundary layer approach (MBLA), the influence of the second term of the asymptotic expansion, i.e., the T-stress, was taken into account. In this case, the displacements on the external edges of the model were determined by the following Equation (4):

(4) $$\begin{array}{c} u_{1}=\frac{K}{E}\left(1+\nu \right)\sqrt{\frac{r}{2\pi }}\cos\left(\frac{\theta }{2}\right)\left[\kappa -1+2\sin^{2}\left(\frac{\theta }{2}\right)\right]+\left(1-\nu^{2}\right)\frac{T}{E}r\cos\left(\theta \right)\\ u_{2}=\frac{K}{E}\left(1+\nu \right)\sqrt{\frac{r}{2\pi }}\sin\left(\frac{\theta }{2}\right)\left[\kappa +1-2\cos^{2}\left(\frac{\theta }{2}\right)\right]-\nu \left(1+\nu \right)\frac{T}{E}r\sin\left(\theta \right) \end{array}$$

where: *κ* = 3–4*ν* for the plane strain condition, *K* is the stress intensity factor, *r* and *θ* are the coordinates of the polar system located at the crack tip.

**Figure 3:**
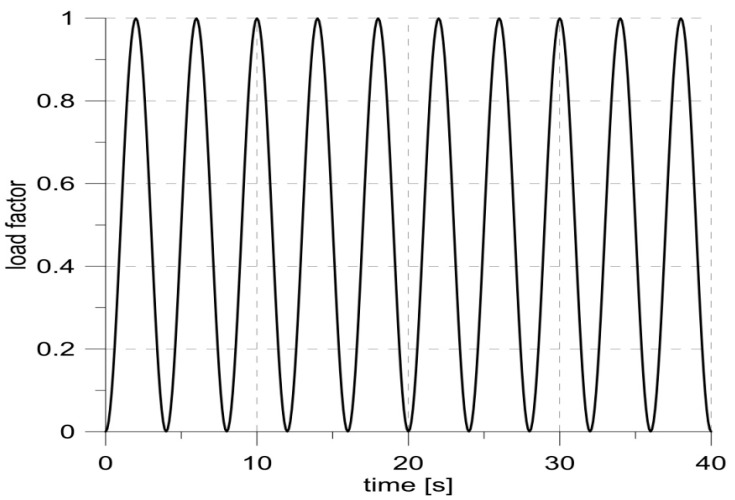
Changes in the load factor describing the amplitude of the loads regarding the maximum load.

The simulation was carried out for 10 cycles of repeating loads according to a sinusoid with an amplitude of 0.5 and a mean value of 0.5 (Figure 3).

To the edge of the semi-circle lying in front of the crack (Figure 2), the cohesive elements were attached (an element-based approach was used). The nodes in this zone were moved, so the initial height of the cohesive zone was equal to zero. Additionally, to improve the stabilization of the model and avoid uncontrolled movements of nodes located in the symmetry plane, their displacements were tied to the displacements of the corresponding nodes connected to the semi-circle nodes.

**Figure 4:**
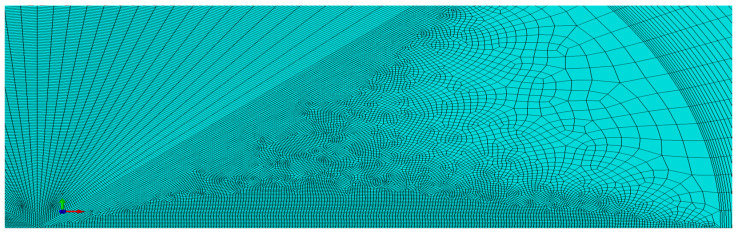
Finite element mesh in the crack tip neighborhood.

The model was filled with 36,464 finite elements. Because the MBLA problem was modeled in a plane strain, 4-node CPE4R elements were used, and along the symmetry line, 350 cohesive elements (COH2D4) were applied. The size of the elements in the crack tip neighborhood was kept constant (Figure 4). The cell size was 0.05 mm for the biggest crack increases (T = 0.0σ_0_ and T = −0.1σ_0_) and 0.01 mm for small crack growths (T = −0.25σ_0_ and T = −0.5σ_0_). A comparison of the results for the two meshes for T = −0.25σ_0_ showed only negligible differences in the obtained results. The size of the elements was at least 5 times smaller than the size of the elements used in the analysis presented in detail in [35,36], where the same material was tested.

The computations were conducted in Abaqus 6.12-2. The Central Processing Unit time in the Linux environment was approximately 22 h, while in the Windows system the same calculations lasted for over 60.5 h.

## 5. Material

The applied material played only a demonstrative role. In the model, high hardening steel for pressure vessel 2.25 Cr 1Mo with the yield strength of 210 MPa, power exponent n = 5, Young’s modulus = 206 GPa, and Poisson’s ratio ν = 0.3 was used [35]. The Huber–Mises yield condition with isotropic hardening was adopted. The cohesive zone was modeled by an element-based approach. The cohesive stress after which the process of degradation of the bonding began was assumed to be equal to 3.83 times the yield stress, i.e., 805 MPa. Linear degradation of the bonding was assumed, while the total degradation of the bonding occurred when a total displacement of 0.03 mm was reached. It was also assumed that the fracture occurred only according to the first mode of loading. The parameters of the cohesive zone were selected to obtain the crack growth for different constraint levels within the zone of fine mesh. For such conditions, the crack growth during simulated loading was small compared to the external radius of the model and not significant from the point of view of MBLA.

## 6. Load Validation

**Figure 5:**
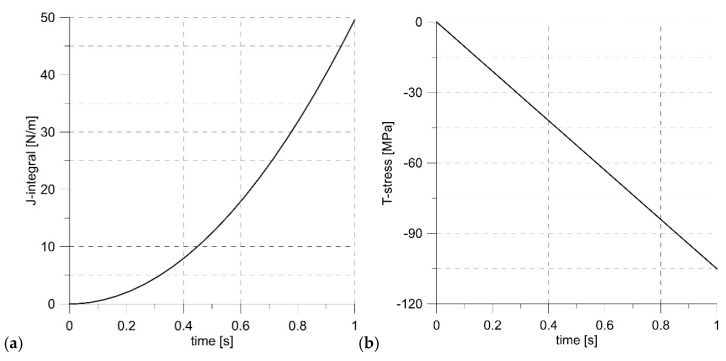
Changes of the J-integral (**a**) and T-stress (**b**) levels.

Since the load was applied to nodes located in the external layer utilizing formulae (4), the numerical model required checking if it gave the correct results. The validation was made by the simulation of the model assuming an elastic material, i.e., assuming Young’s modulus E = 206 GPa and Poisson’s ratio ν = 0.3. A linearly increasing load was applied to the edges of the model with ΔK = 106 MPa√m (J_input_ = 49.55 N/m) and T-stress T_input_ = −105.11 MPa. As a result, the values J_output_ = 49.56 N/m and T_output_ = −105.12 MPa were obtained (Figure 5), which showed that the structure of the numerical model did not affect the results obtained.

## 7. Results

**Figure 6:**
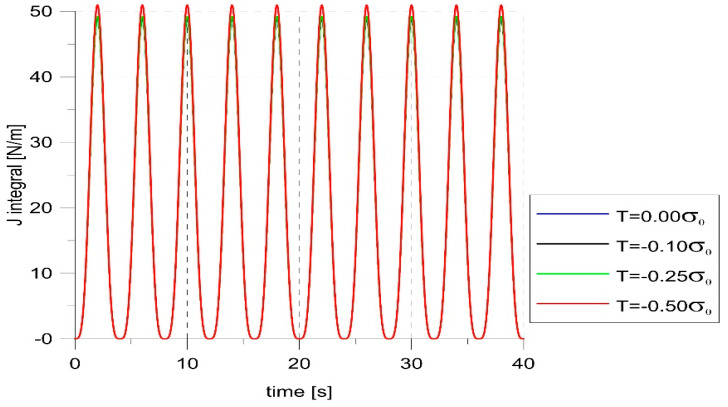
J-integral changes for different constraint levels.

The most reasonable way to express the crack tip load was the J-integral obtained by default in ABAQUS. The graph showing changes in the J-integral for various levels of constraints is presented in Figure 6. An insignificant influence of the T-stress value on the values of the J-integral can be seen. Obtained J-integral values at maximum load slightly increased with a decreasing level of the constraint, but all values fit in the range 49.5 N/m ± 2%. Figure 6 also proves that the small crack increment that occurred during loading did not affect the level of the crack tip load. It remained constant, as shown in the maxima of the J-integral.

**Figure 7:**
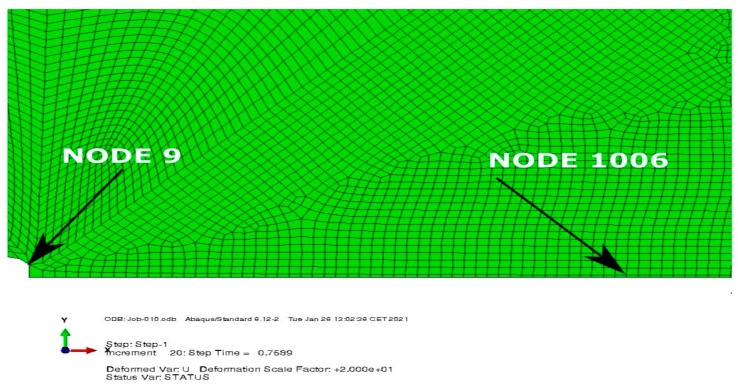
The location of the nodes used in the analysis.

The crack opening displacement (COD) and opening stress of the two nodes were analyzed (Figure 7).

Node number 9 lay at the initial crack tip position and node 1006 lay at the location of the crack tip for T = −0.1σ_0_, behind the crack tip, at the end of the simulation. In that way, for the highest level of constraint, node 1006 lay on a newly created edge, while for T = −0.25σ_0_ and T = −0.5σ_0_ node 1006 was located in a weak zone between the crack tip and the maximum of opening stress. In the case of finer mesh, the equivalent node that was located at the same position had the number 6.

**Figure 8:**
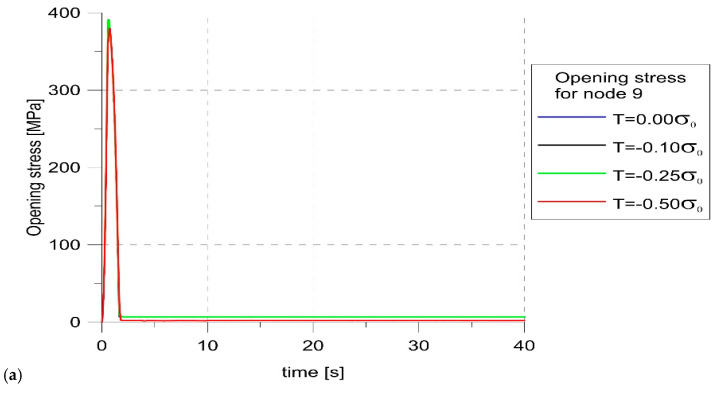

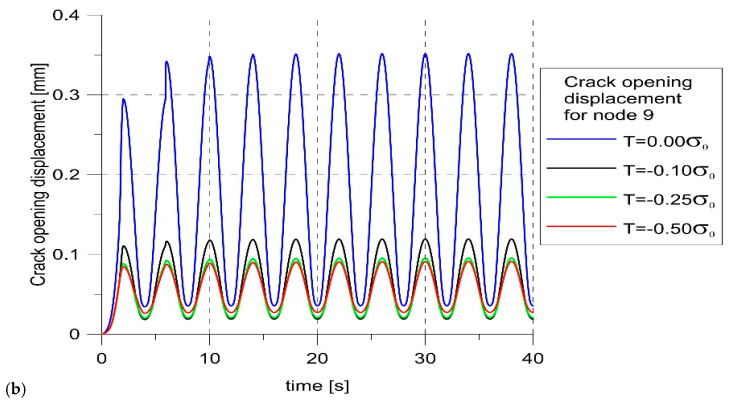
Changes in the opening stress (**a**) and crack opening displacement (COD (**b**) with load for node 9.

Figure 8 showed changes in the opening stress and COD for node 9. From Figure 8, it followed that the crack in this node location was open throughout the entire load period (positive COD values), and for all load cases, this node was released during the first load cycle (the opening stress was greater than zero only for the first cycle). The amount of COD depended on the level of constraints, and beyond the first cycle, it could be seen that the maximum values were recorded for the highest levels of constraints.

**Figure 9:**
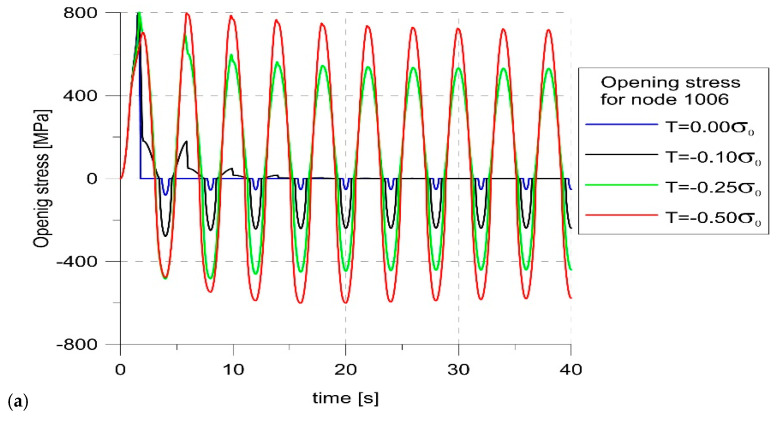

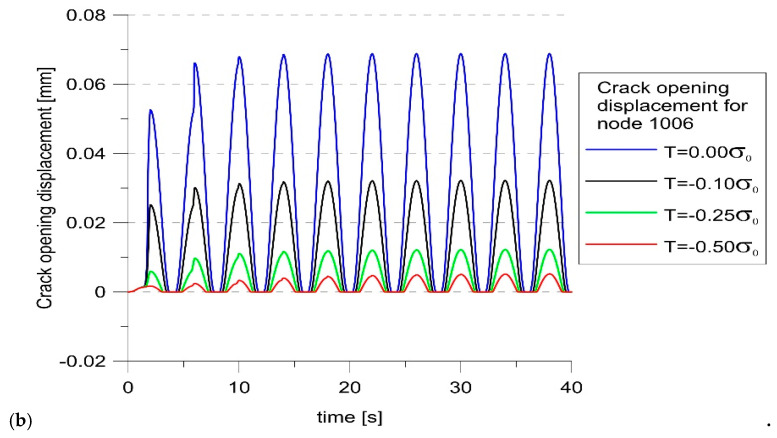
Changes in the opening stress (**a**) and COD (**b**) with load for node 1006.

As the crack increased in the first cycle of loading, regardless of the level of constraints, the node located in front of the crack at the last loading cycle for the constraint level T = −0.1σ_0_ was also analyzed (Figure 9). Figure 9a shows that, unlike in the case of node 9, negative opening stress appeared, indicating closure of the crack [37]. This was confirmed by the picture of changes in COD (Figure 9b). The crack opening at node 1006 occurred earlier for higher levels of constraints. For T-stress equal to zero, the crack propagated beyond node 1006 already in the first cycle, while for T = −0.1σ_0_, the crack tip reached this point only at the 5th cycle. For lower levels of constraint, the actual crack tip did not propagate to this point; however, point 1006 was located in the area between the actual crack tip and the fictitious crack tip. Moreover, the size of the crack opening depended very clearly on the level of constraint. In the case of a high level of constraints, when the crack grew the fastest and node 1006 was outside the crack tip zone, it could be seen that the COD for T = 0 was about 50% bigger compared to the opening for T = −0.1σ_0_.

**Figure 10:**
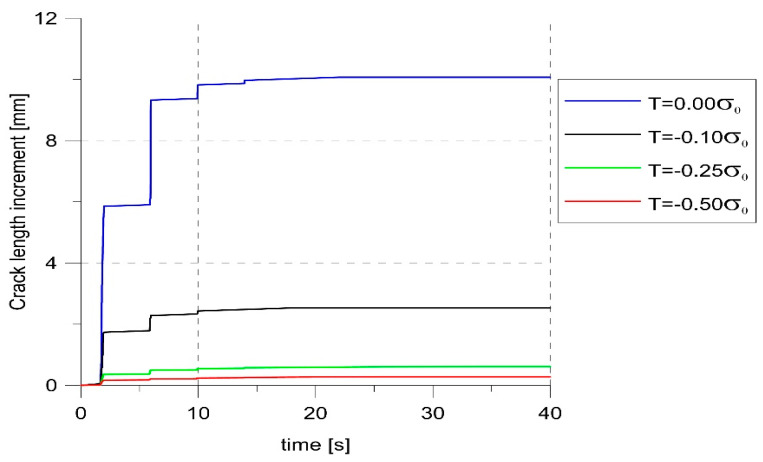
The crack increase.

**Figure 11:**
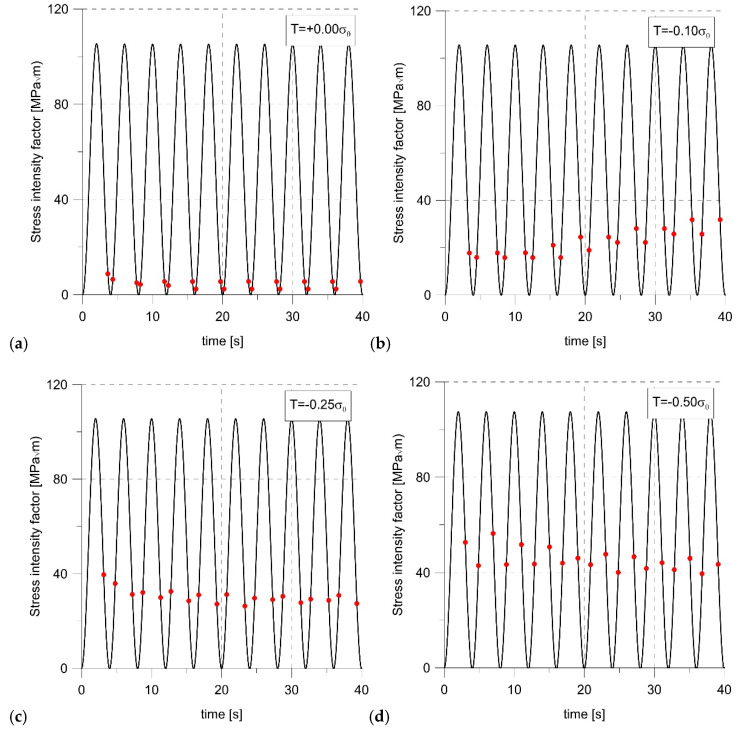
The stress intensity factor (SIF)_opening_ values for subsequent loading cycles for: T = +0.25σ_0_ (**a**), T = +0.00σ_0_ (**b**), T = −0.50σ_0_ (**c**), and T = −0.75σ_0_ (**d**).

The constraints not only affected the level of the opening stress and the size of the opening. As can be seen in Figure 10, the highest crack growth rate was obtained for T = 0. The lower the constraint level, the shorter crack increase was obtained. To determine the moment of crack opening at each load cycle, the values of the opening stress for node 1006 were analyzed. If it changed from negative to positive, an opening occurred. On this basis, the moments of opening and closing of the crack at different levels of constraints were determined and marked with dots on the load diagrams (Figure 11). Comparing the values of SIF at the moment of opening, it was clear that these values were strongly influenced by the constraint level. For the highest level of constraint, the crack opened earlier.

**Figure 12:**
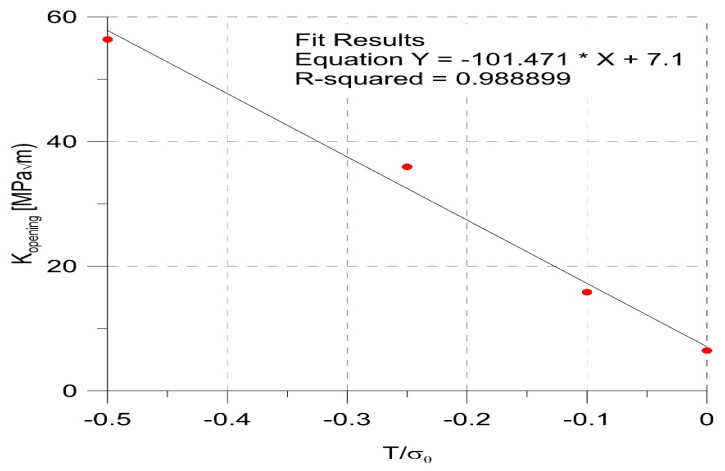
Relationship between the SIF_opening_ values and the level of in-plane constraints.

The relationship between the value of the opening SIF for the second loading cycle and the level of constraints was so regular (Figure 12) that it could be described with high accuracy by the linear Equation (5):

(5) $$K_{opening}=-101.471\frac{T}{\sigma_{0}}+7.1$$

where *σ*_0_ is the yield stress. As a result, the effective range of SIF during fatigue loading will be smaller for low constraint geometries, as in such geometries, the crack growth under the same loading should be slower.

## 8. Conclusions

**Figure 13:**
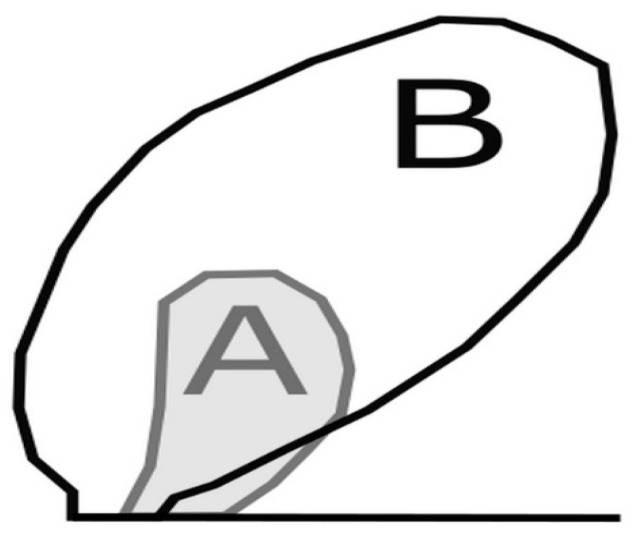
Comparison of plastic zones at the first load maximum for T = 0σ_0_ (**A**), and T = −0.5σ_0_ (**B**).

In the paper, the numerical model of a crack subjected to cyclic loading was applied. The obtained results, to some extent, were compared with the analytical solution. Satisfactory convergence of the results with theoretical formulae suggested that the model predicted the behavior of a crack subjected to cyclic loading at different levels of in-plane constraint at least qualitatively well. The influence of the level of in-plane constraints on the behavior of the numerically modeled crack under fatigue load was clear. The boundary conditions corresponding to different T-stress levels caused changes in the growth rate in such a way that the crack increased faster for higher levels of constraints. Two factors influenced this. First, the lower the level of constraint was, the larger the plastic zones develop in the neighborhood of the crack tip (Figure 13). Absorbing energy through plastic zones meant that less energy was used to create a new surface. The second factor was the influence of constraints on the closure of the crack. The lower the level of constraint, the longer the crack under load remained closed, and the lower the effective SIF. As a result, the low level of in-plane constraint caused the fracture growth to slow down significantly.

Research results indicated that it was worth undertaking experimental research on the problem of the effect of in-plane constraints on a fatigue crack growth rate and fatigue crack closure, which may lead to the creation of a universal law of crack growth, in which the influence of geometry could be included by using one, easily determinable parameter. The results of experimental investigations presented for various geometries did not use a common parameter to describe the influence of in-plane geometry, so these are hard to compare. Application of T-stress or Q-parameter should benefit from an easy comparison of the results obtained for a selected material, prediction of fatigue crack behavior for different shapes of machine members, and, finally, obtain closed formulae to describe the fatigue process.

To fully accomplish the objective of the paper, the influence of in-plane constraints on crack behavior should be described in a quantitative way. This will be obtained by the experimental program in which several geometries described by different levels of in-plane constraint will be subjected to the same value of cyclic load defined by the stress intensity factor or J-integral.

## Data Availability

No new data were created or analyzed in this study. Data sharing is not applicable to this article.

## Nomenclature

E	Young’s modulus
J_I_	J-integral
K_I_	stress intensity factor
T-stress	second term of the asymptotic expansion
r	radial coordinate of the polar system located at the crack tip
u_i_	components of the displacement vector
σ_0_	yield stress
θ	tangential coordinate of the polar system located at the crack tip
ν	Poisson’s ratio
